# Protection from cigarette smoke‐induced vascular injury by recombinant human relaxin‐2 (serelaxin)

**DOI:** 10.1111/jcmm.12802

**Published:** 2016-02-24

**Authors:** Alessandro Pini, Giulia Boccalini, Maria Caterina Baccari, Matteo Becatti, Rachele Garella, Claudia Fiorillo, Laura Calosi, Daniele Bani, Silvia Nistri

**Affiliations:** ^1^ Department of Experimental & Clinical MedicineSection of Anatomy & Histology & Research Unit of Histology & EmbryologyUniversity of FlorenceFlorenceItaly; ^2^ Section of PhysiologyUniversity of FlorenceFlorenceItaly; ^3^ Department of Experimental & Clinical Biomedical Sciences ‘Mario Serio’Section of BiochemistryUniversity of FlorenceFlorenceItaly

**Keywords:** relaxin, aorta, cigarette smoke, oxidative stress, eNOS

## Abstract

Smoking is regarded as a major risk factor for the development of cardiovascular diseases (CVD). This study investigates whether serelaxin (RLX, recombinant human relaxin‐2) endowed with promising therapeutic properties in CVD, can be credited of a protective effect against cigarette smoke (CS)‐induced vascular damage and dysfunction. Guinea pigs exposed daily to CS for 8 weeks were treated with vehicle or RLX, delivered by osmotic pumps at daily doses of 1 or 10 μg. Controls were non‐smoking animals. Other studies were performed on primary guinea pig aortic endothelial (GPAE) cells, challenged with CS extracts (CSE) in the absence and presence of 100 ng/ml (17 nmol/l) RLX. In aortic specimens from CS‐exposed guinea pigs, both the contractile and the relaxant responses to phenylephrine and acetylcholine, respectively, were significantly reduced in amplitude and delayed, in keeping with the observed adverse remodelling of the aortic wall, endothelial injury and endothelial nitric oxide synthase (eNOS) down‐regulation. RLX at both doses maintained the aortic contractile and relaxant responses to a control‐like pattern and counteracted aortic wall remodelling and endothelial derangement. The experiments with GPAE cells showed that CSE significantly decreased cell viability and eNOS expression and promoted apoptosis by sparkling oxygen free radical‐related cytotoxicity, while RLX counterbalanced the adverse effects of CSE. These findings demonstrate that RLX is capable of counteracting CS‐mediated vascular damage and dysfunction by reducing oxidative stress, thus adding a tile to the growing mosaic of the beneficial effects of RLX in CVD.

## Introduction

Smoking is regarded as one of the major risk factors for the development of cardiovascular diseases (CVD), accounting for 80% increased risk for coronary artery insufficiency in smokers compared with non‐smokers 1. Recently, the World Health Organization (WHO) predicted that, by 2030, deaths related to cigarette smoke (CS) will increase up to eight million individuals per year worldwide 2. This trend is expected to increase in parallel with the earlier onset of tobacco use. Furthermore, clinical studies have shown that young smokers have a higher risk to develop vascular functional and histological abnormalities which are early hallmarks of atherosclerosis 3, 4. Hence, there is a major interest in the development of new therapeutic strategies that can prevent or reduce the adverse effects of CS on the cardiovascular system. Increasing evidence supports the knowledge that oxidative stress and inflammation provide the pathophysiological link between cigarette smoking and CVD 5, 6, 7, 8, 9, 10. Endothelial dysfunction is one of the earliest pathological events that occur in the development of CVD 7. Several clinical and experimental studies indicate that CS oxidants can directly impair endothelial function, primarily through a lack of nitric oxide bioavailability which results both from decreased nitric oxide formation by the dysfunctional endothelium and increased nitric oxide scavenging by oxygen free radicals 10, 11, 12, 13. There is conflicting evidence regarding regulation of endothelial nitric oxide synthase (eNOS) by CS extract (CSE) at the transcriptional/translational level in cellular models with some suggesting reduced eNOS mRNA/protein levels 14, 15, 16 while others demonstrating unchanged 17 or even increased eNOS levels 18: the latter possibly likely represents an attempt of endothelial cells to compensate for eNOS impairment. These functional alterations are accompanied by histological changes in vascular wall consisting in increase in the intima‐media wall thickness, a marker of early atherosclerosis, 4, 18, 19, elastic fibres damage, cytoplasmic vacuolation of smooth muscle cells of the intima‐media, and endothelial loss caused by disruption of the adhesion between endothelial cells and their basement membrane 20, 21.

On the above grounds, we designed the present study to investigate whether serelaxin (RLX), the recombinant molecule corresponding to the human hormone relaxin‐2 22, shown to possess promising therapeutic properties in cardiac diseases 23, 24, may be credited of a protective effect against CS‐induced vascular damage and dysfunction. This working hypothesis is based on the following mainstays: (*i*) blood vessels are a physiological target of RLX and their cells express the specific RLX receptor RXFP1 25; (*ii*) RLX up‐regulates NOS expression and nitric oxide production in vascular endothelial cells 26, 27, 28, 29, 30; (*iii*) RLX improves inflammation‐induced endothelial dysfunction and NOS fall 31, 32; and (*iv*) RLX reduces oxidative stress and nitric oxide failure in different animal models of vascular injury 33, 34, 35, 36, 37.

## Materials and methods

### Reagents

Serelaxin was kindly provided by the RRCA Relaxin Foundation (Florence, Italy); its use in the present animal model is supported by the recent finding that human relaxin can bind to RXFP1 relaxin receptor of various species, including the guinea pig 38. Acetylcholine was from Sigma Chemical (St. Louis, MO, USA), phenylephrine from Tocris Bioscience (Bristol, UK). Kentucky Reference cigarettes 3R4F, each containing 11 mg of total particulate matter, 9.4 mg of tar and 0.73 mg of nicotine, were obtained from the Kentucky Tobacco Research Council (Lexington, KY, USA). Unless otherwise specified, the other reagents used for the experiments were from Sigma‐Aldrich (Milan, Italy).

### *In vivo* study

#### Exposure of guinea pigs to CS

Male Hartley albino guinea pigs weighing 300–350 g were used for the experiments (Harlan, Correzzana, Italy). Animal handling and use complied with the European Community guidelines for animal care (2010/63/EU) and were approved by the Committee for Animal Care and Experimental Use of the University of Florence. The animals were housed on a 12 hrs light/dark cycle at 22°C room temperature and had free access to food and water. The experiments were designed to minimize pain and the number of animals used. Sacrifice was carried out by decapitation.

The animals were divided into the following experimental groups (*n* = 6/group):

Group 1: Control untreated animals;
Group 2: Animals exposed daily to CS for 8 weeks;
Group 3: Animals exposed daily to CS for 8 weeks and treated with RLX given by continuous subcutaneous (s.c.) infusion using osmotic minipumps (Alzet; DURECT Corporation, Cupertino, CA, USA). The pumps were implanted 1 day before starting the exposure to CS on the back upon anaesthesia (i.p. injection of ketamine hydrochloride, 100 mg/kg b.w. and xylazine, 15 mg/kg b.w.) and filled to deliver a daily dose of 1 μg for the whole duration of CS exposure;
Group 4: Animals exposed to CS and treated with RLX given by minipumps as above, but delivering a daily RLX dose of 10 μg for the whole duration of CS exposure.

The animals were subjected to CS exposure in a smoke chamber, according to Das *et al*. 39 with minor modifications. The smoke chamber (2.5 l) was similar to a vacuum desiccator equipped with an open tube for cigarette positioning at one end and a vacuum‐connected tube and stopcock at the opposite end. To each group of CS‐exposed animals, five 3R4F reference cigarettes were administered daily. Each cigarette was fitted on the inlet tube and lit; then, a puff of CS was introduced into the chamber containing the animals by applying a mild suction of 4 cm water for 20 sec. The guinea pigs were exposed to the accumulated smoke for further 40 sec., for a total duration of CS exposure of 60 sec. After a pause of 60 sec. during which the chamber was opened and ventilated with fresh air, a second puff was administered with the same procedure. The gap between each of the 5 cigarettes/day was 1 hr. At the end of the treatment, the animals were anaesthetized by i.p. injection of ketamine hydrochloride (100 mg/kg b.w.) and xylazine (15 mg/kg b.w.), blood and tissue samples were collected and processed for functional, morphological and biochemical analyses.

#### Determination of serum RLX levels

The circulating RLX levels were determined in guinea pig serum by ELISA (R&D Systems, Minneapolis, MN, USA) according to the manufacturer's instructions.

#### Functional studies

Segments of thoracic aortas were cut into helical strips (3 mm width, 20 mm length) and connected to a force displacement transducer (Grass FT03, Grass Technologies, Middleton, WI, USA) coupled to a polygraph (Grass 7K, Grass Technologies) for continuous recording of isometric tension. Strips were mounted in 5‐ml organ baths containing Krebs–Henseleit solution, gassed with 95% O_2_–5% CO_2_ mixture, of the following composition (mM): NaCl 118, KCl 4.7, MgSO_4_ 1.2, KH_2_PO_4_ 1.2, NaHCO_3_ 25, CaCl_2_ 2.5 and glucose 10 (pH 7.4) and kept at 37°C. Preparations were allowed to equilibrate for 1 hr under an initial load of 2.5 g, as previously reported 40. In each experimental group, the contractile responses to phenylephrine (2 × 10^−7^ M) were evoked. When contraction reached a stable plateau phase, acetylcholine (Ach, 2 × 10^−6^ M) was added. The chosen concentrations are similar to those previously used for similar purposes 40, 41. The interval between two subsequent applications of phenylephrine was ≥15 min., during which repeated washes with Krebs–Henseleit solution were performed.

#### Morphological studies

Aortic tissue samples taken at sacrifice were fixed by immersion in 3% paraformaldehyde, dehydrated in graded ethanol, embedded in paraffin and cut in 5‐μm thick sections. Histological slides were stained with haematoxylin and eosin. Other tissue samples were fixed in 4% glutaraldehyde and 1% osmium tetroxide and embedded in Epon 812. Ultrathin sections were stained with uranyl acetate and alkaline bismuth subnitrate and examined under a JEM 1010 electron microscope (Jeol, Tokyo, Japan) at 80 kV.

#### Immunohistochemical detection of eNOS

Endothelial nitric oxide synthase immunoreactivity was determined on paraformaldehyde‐fixed, paraffin‐embedded sections incubated with rabbit polyclonal anti‐eNOS (1:100, 4°C overnight; Abcam, Milan, Italy) and Alexa‐fluor 568‐labelled goat anti‐rabbit IgG (1:300, 1 hr at room temperature; Invitrogen, Milan, Italy). Negative controls were carried out by omitting the primary antiserum.

#### Determination of 8‐hydroxy‐2′‐deoxyguanosine

8‐hydroxy‐2′‐deoxyguanosine (8‐OHdG), an indicator of oxidative DNA damage, was measured in guinea pig serum with high sensitivity 8‐OHdG Check ELISA kit (IMKOGHS, Jaica, Japan), according to the manufacturer's instructions. Serum samples were passed through an ultrafilter with a 10 kD molecular weight cut‐off (YM‐10; Millipore Co., Bedford, MA, USA) to remove any large molecular weight substances and then used for 8‐OHdG determination.

#### Detection of free carbon monoxide in plasma

Free carbon monoxide (CO) was measured in the plasma of the animals of the different experimental groups as an index of the degree of exposure to CS. The amount of free CO in plasma was measured with a gaseous CO detector (RGA3, Reduction Gas Analyzer; SAES Getters, Milan, Italy) as described 42. Measurements were obtained by comparison with a CO standard curve prepared immediately before analysis and expressed as parts per million.

### *In vitro* study

#### Cigarette smoke extracts preparation

Cigarette smoke extracts solution was prepared by bubbling smoke from two 3R4F cigarettes, 30 sec. each, in 50 ml of PBS according to Niu *et al*. 43, with minor modifications. The resulting solution, assumed as 100% CSE, was adjusted to pH 7.4, and filtered through a 0.2 μm filter (Millipore Co.). Cigarette smoke extracts solution was freshly prepared on the day of the experiment and immediately used.

To establish the effective dose of CSE, preliminary concentration‐ and time‐dependent studies were performed (data not shown). Guinea pig aortic endothelial cells were exposed to 1–15% CSE for 4–12 hrs and cell viability was evaluated by trypan blue. Accordingly, 10% CSE solution (vol/vol) for 4 hrs was used in all the experiments because it gave a well‐appreciable toxic effect without causing massive cell death.

#### Isolation and culture of guinea pig aortic endothelial cells

Male Hartley albino guinea pigs (Harlan), 2‐ to 3‐month old, were anaesthetized by i.p. injection of ketamine hydrochloride (100 mg/kg b.w.) and xylazine (15 mg/kg b.w.) and killed by decapitation. The thoracic aorta was quickly removed and guinea pig aortic endothelial (GPAE) cells were isolated based on a previously described method 44.

Characterization of the isolated GPAE cells was performed by immunocytochemistry and cytochemistry, as previously reported 27. The percentage of immunoreactive cells for endothelial markers, such as vimentin and von Willebrand factor ranged between 96% and 98% (data not shown). For all experiments, cells were used at the first and second passage.

Guinea pig aortic endothelial cells were pre‐treated or not with RLX 100 ng/ml (17 nmol/l) for 24 hrs and then exposed to CSE for 4 hrs. This RLX dose was chosen because it was shown to induce significant cell protection from oxidative stress 45. Control cultures not exposed to CSE were also prepared.

#### Total RNA extraction and RT‐PCR

Expression levels of mRNA for RXFP1, eNOS and β‐actin were assayed by RT‐PCR in GPAE cells. One microgram of total RNA, extracted with TRIzol Reagent (Invitrogen), was reverse transcribed and amplified with SuperScript One‐Step RT‐PCR System (Invitrogen). After cDNA synthesis for 30 min. at 55°C, the samples were pre‐denatured for 2 min at 94°C and then subjected to 35 cycles of PCR performed at 94°C for 15 sec., alternating with 55°C for 30 sec. (RXFP‐1 and β‐actin) or 57°C for 30 sec. (eNOS) and 72°C for 1 min.; the final extension step was performed at 72°C for 5 min. The following guinea pig gene‐specific primers were used: RXFP1 (XM_003476825.2), forward 5′‐GGT TGC TTG GTT GGT TCT GT‐3′ and reverse 5′‐ACG TTG GGA GGG AGT TTT CT‐3′; eNOS (NM_001172985), forward 5′‐CCT GCT TCA TTA GAG GGG CT‐3′ and reverse 5′‐TCG ACA GCC AAA CAC CAA AG‐3′; β‐actin (NM_001172909.1), forward 5′‐CTT TGC TGC GTT ACA CCC TT‐3′ and reverse 5′‐ATG CTT GCT CCA ACC AAC TG‐3′. PCR products were electrophoresed on a 2% agarose gel and stained with ethidium bromide.

#### Trypan blue viability assay

The trypan blue exclusion method was used to assess cell viability. Guinea pig aortic endothelial cells (5 × 10^4^/well) were seeded in 24‐well plates. At the end of the treatments, the cells were gently detached by trypsin/ethylenediaminetetraacetic acid (EDTA), resuspended in culture medium and mixed 1:1 with 0.4% trypan blue solution. The final cell suspensions were counted under a phase contrast inverted microscope using a Burker chamber. Viable cells were expressed as percentage of the total counted cells.

#### Mitochondrial membrane potential

Mitochondrial membrane potential (Δψ) was assessed as previously reported 46 using tetramethylrhodamine methyl ester perchlorate (TMRM), a lipophilic potentiometric fluorescent dye that distributes between mitochondria and cytosol in proportion to Δψ by virtue of its positive charge. Thus, the fluorescence intensity depends on dye accumulation in mitochondria, which is directly related to Δψ. For confocal microscope analysis, GPAE cells were cultured on glass coverslips and loaded for 20 min. at 37°C with TMRM, dissolved in 0.1% dimethylsulfoxide (DMSO) to a 100 nM final concentration in the culture medium. The cells were fixed in 2% paraformaldehyde for 10 min. at room temperature and the TMRM fluorescence analysed under a confocal Leica TCS SP5 laser scanning microscope (Mannheim, Germany) with 543‐nm excitation wavelength and ×63 oil immersion objective. Δψ was also quantified by flow cytometry: single‐cell suspensions were incubated for 20 min. at 37°C in the dark with TMRM dissolved in M199 medium (100 nM) and then analysed using a FACSCanto flow cytometer (Becton‐Dickinson, San Jose, CA, USA).

#### Assessment of Caspase‐3 activity

Guinea pig aortic endothelial cells seeded on glass coverslips were incubated with FAM‐FLICA^™^ Caspase 3&7 assay kit (Immunochemistry Technologies, Bloomington, MN, USA) for 30 min. as previously reported 47 and then fixed in 2% paraformaldehyde for 10 min. at room temperature. Fluorescence was detected by a confocal Leica TCS SP5 confocal laser scanning microscope with 488‐nm excitation wavelength and ×63 oil immersion objective. Caspase‐3 activity was also quantified by flow cytometry: single‐cell suspensions were incubated with FAM‐FLICA^™^ for 30 min. at 37°C and then analysed using a FACSCanto flow cytometer (Becton‐Dickinson).

#### Determination of intracellular ROS

Guinea pig aortic endothelial cells seeded on glass coverslips were loaded with the ROS‐sensitive fluorescent probe 2′,7′‐dichlorodihydrofluorescein diacetate (H_2_DCFDA; Invitrogen, 2.5 μmol/l) – dissolved in 0.1% DMSO and Pluronic acid F‐127 (0.01% w/v) – added to cell culture media for 15 min. at 37°C 48. The cells were fixed in 2% paraformaldehyde for 10 min. at room temperature and the H_2_DCFDA fluorescence analysed using a Leica TCS SP5 confocal laser scanning microscope with 488‐nm excitation wavelength and ×63 oil immersion objective. Intracellular ROS levels were also monitored by flow cytometry: single‐cell suspensions were incubated with H_2_DCFDA (1 μmol/l) for 15 min. at 37°C and then analysed using a FACSCanto flow cytometer (Becton‐Dickinson).

#### Evaluation of lipid peroxidation

Lipid peroxidation was investigated by confocal scanning microscopy using BODIPY 581/591 C11 (Life Technologies, Monza, Italy), a lipophilic fluorescent probe that mimics the properties of natural lipids 49. BODIPY acts as a fluorescent lipid peroxidation reporter as it shifts its fluorescence from red to green in the presence of oxidizing agents. Guinea pig aortic endothelial cells cultured on glass coverslips were loaded with BODIPY, dissolved in 0.1% DMSO (2 μM final concentration in the culture medium), for 30 min. at 37°C. The cells were fixed in 2% paraformaldehyde for 10 min. at room temperature and analysed using a Leica TCS SP5 confocal laser scanning microscope with 581 nm excitation wavelength and ×63 oil immersion objective. Lipid peroxidation was also quantified by flow cytometry: single‐cell suspensions were incubated, in the dark, for 30 min. at 37°C with BODIPY (1 μM) in cell culture medium and then analysed using a FACSCanto flow cytometer (Becton‐Dickinson).

All the flow cytometry data were analysed using FACSDiva software (Becton‐Dickinson).

#### Western blotting

After treatments, GPAE cells were lysed in cold buffer (10 mM Tris/HCl pH 7.4, 10 mM NaCl, 1.5 mM, MgCl_2_, 2 mM Na_2_ EDTA, 1% Triton X‐100), added with 10× Sigmafast Protease Inhibitor cocktail tablets. Total protein content was measured spectrophotometrically using micro‐BCA^™^ Protein Assay Kit (Thermo Fisher‐Pierce, Waltham, MA, USA). Forty microgram of total proteins were electrophoresed by SDS‐PAGE and blotted onto nitrocellulose membranes (Amersham, Cologno Monzese, Italy). The membranes were incubated overnight at 4°C with rabbit polyclonal anti‐eNOS (1:1000; Abcam) and rabbit polyclonal anti‐β‐actin antibodies (1:20,000; Sigma‐Aldrich), assuming β‐actin as control invariant protein. Specific bands were detected using rabbit peroxidase‐labelled secondary antibodies (1:15,000; Vector, Burlingame, CA, USA) and enhanced chemiluminescent (ECL) substrate (Bio‐Rad, Milan, Italy).

#### Data analysis and statistical tests

The reported data are expressed as the mean ± S.E.M. of six animals per group or at least three independent cell culture experiments. Statistical comparison of differences between groups was carried out using one‐way anova followed by Student–Newman–Keuls multiple comparison test. A *P*‐value ≤0.05 was considered significant. GraphPad Prism 2.0 statistical program (GraphPad Software, San Diego, CA, USA) was used for statistical analysis.

## Results

### Plasma CO levels

The animals of the different experimental groups subjected to chronic CS had significantly elevated CO levels (CS‐exposed: 37.9 ± 3.2; CS+RLX 1 μg/day: 32.1 ± 3.1; CS+RLX 10 μg/day: 37.5 ± 5.8) compared with the controls (6.4 ± 0.8: *P* < 0.001 *versus* the other groups). No significant differences were detected among the CS‐exposed groups, suggesting that all the animals were subjected to the same level of CS‐induced toxicity.

### Plasma RLX levels

The circulating levels of RLX evaluated at the end of the experiment were 308 ± 44 pg/ml and 2.5 ± 0.6 ng/ml upon 1 and 10 μg daily doses respectively. The values measured in the untreated controls and the CS‐exposed animals were consistently below the detection threshold.

### *Ex vivo* aortic contractility

**Figure 1:**
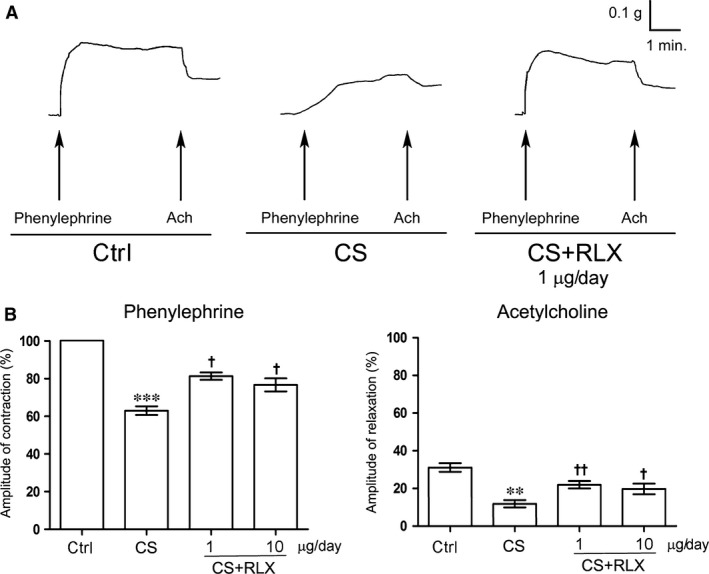
*Ex vivo* contractility of aortic strips from guinea pigs of the different experimental groups. (**A**) Typical tracings in response to phenylephrine (2 × 10^−7^ M) and acetylcholine (Ach, 2 × 10^−6^ M). In the CS‐exposed animals, the amplitude and slope of both responses are reduced as compared with the untreated controls. RLX reverted the tracing amplitude and slope to a pattern similar to that of the controls. (**B**) Mean amplitude of contractile and relaxant responses to phenylephrine and Ach, respectively, in the different experimental groups. Amplitudes of the contractile responses to phenylephrine and the relaxant responses to Ach are expressed as per cent increase and decrease, respectively, of the maximal contraction evoked by 2 × 10^−7^ M phenylephrine, assumed as 100%. Values are means ± S.E.M. (one‐way anova test), *n* = 6. **P < 0.01, ***P < 0.001 *versus* controls; ^†^ *P* < 0.05 and ^††^ *P* < 0.01 *versus* CS‐exposed animals.

In aortic strips from the untreated control animals, addition of phenylephrine to the bath medium caused a rapidly arising contraction that reached a plateau phase (mean amplitude 0.20 ± 0.05 g) (Fig. 1). In phenylephrine‐precontracted preparations, Ach caused a fast relaxation (Fig. 1) that persisted until washout. In aortic strips from the CS‐exposed guinea pigs, both the contractile and the relaxant responses to phenylephrine and Ach were significantly reduced in amplitude and delayed (Fig. 1). In strips from the CS‐exposed animals treated with RLX at both doses, the amplitude of responses to both phenylephrine and Ach was significantly enhanced as compared with the CS‐exposed animals (Fig. 1). No significant differences were observed between 1 and 10 μg/day RLX.

### Morphology

**Figure 2:**
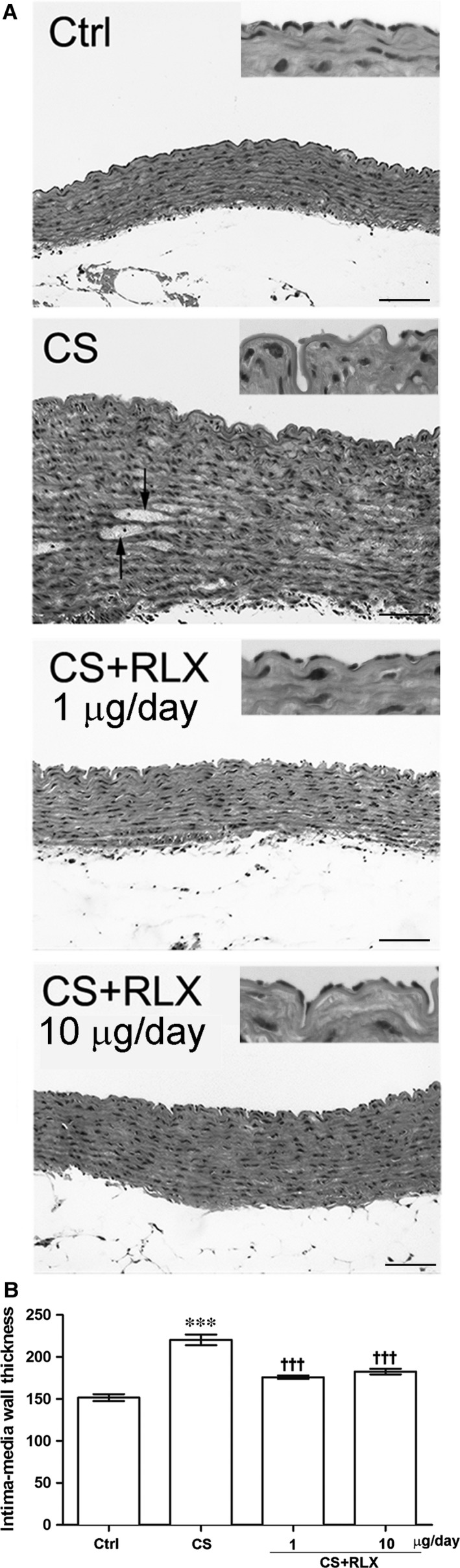
Histology of aortas from guinea pigs of the different experimental groups. (**A**) Representative pictures of aortic transverse sections: the control shows continuous endothelial lining and a tunica media made up of alternate layers of elastic lamellae and smooth muscle cells; CS shows extended loss of endothelial cells, marked thickening of the tunica media with disarrangement of smooth muscle cells and accumulation of amorphous material (arrows); RLX treatment at both doses shows features similar to the control. Insets: details of the aortic intima showing the presence or absence of the endothelial lining. (**B**) Morphometric analysis of aortic wall thickness (intima+media): values are means ± S.E.M. (one‐way anova test), *n* = 6. ***P < 0.001 *versus* controls; ^†††^ *P* < 0.001 *versus* CS‐exposed animals. Haematoxylin & eosin, magnification ×200, bars = 100 μm.

**Figure 3:**
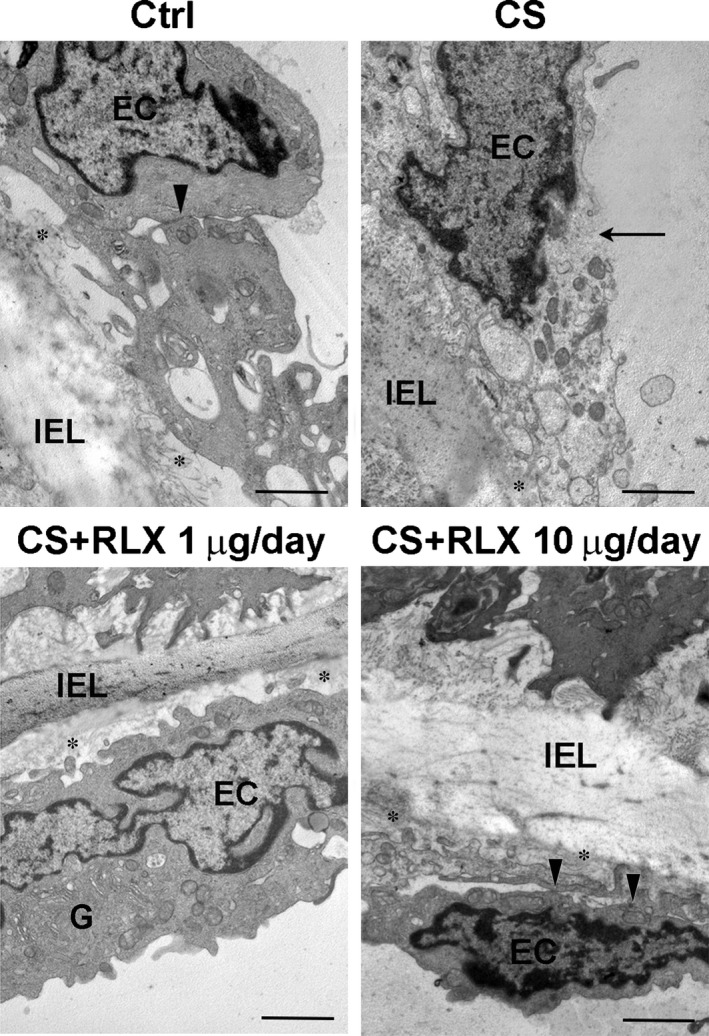
Representative electron micrographs of aortic intima from guinea pigs of the different experimental groups. The control shows normal features of endothelial cells (EC), which are bound by tight junctions (arrowhead); CS exposure causes diffuse endothelial cell vacuolation and plasma membrane rupture (arrow); RLX at both doses results in a normal appearance of the endothelial cells. G: well‐developed Golgi apparatus; asterisks, tracts of basement membrane; IEL, inner elastic lamina; magnification ×6000, bars = 1 μm.

Histological examination of transverse sections of aorta (Fig. 2A) showed that the control guinea pigs had thin wall, showing an intact structure with continuous endothelial lining and alternate layers of elastic lamellae and smooth muscle cells in the tunica media. Cigarette smoke exposure caused extended loss of endothelial cells, significant wall thickening, disarrangement of smooth muscle cells in the tunica media, some of which showed cytoplasmic vacuolation, and accumulation of amorphous material. Conversely, treatment with RLX at both doses during CS exposure resulted in an almost complete disappearance of the noted aortic wall abnormalities. The RLX‐induced reduction of aortic wall thickening caused by CS exposure was confirmed by the morphometric analysis (Fig. 2B). Ultrastructural examination (Fig. 3) revealed that, compared with the controls, CS exposure caused diffuse endothelial cell vacuolation and signs of cell demise, such as plasma membrane rupture and detachment from the other cells and the basement membrane; RLX at both doses prevented these degenerative changes.

### Endothelial cell viability

**Figure 4:**
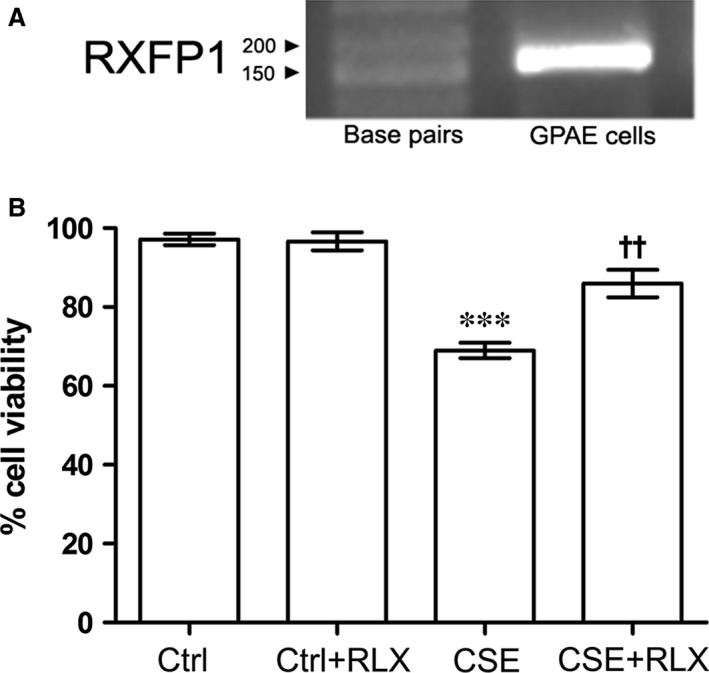
(**A**) RT‐PCR showing the expression of the RLX receptor RXFP1 by GPAE cells. (**B**) Trypan blue viability test performed on GPAE cells: compared with the controls, exposure to CSE causes a significant decrease in cell viability, while pre‐treatment with RLX significantly reduced this effect. RLX *per se* has no effect on cell viability. Values are means ± S.E.M. (one‐way anova test), *n* = 6. ***P < 0.001 *versus* controls; ^††^ *P* < 0.01 *versus* CSE.

**Figure 5:**
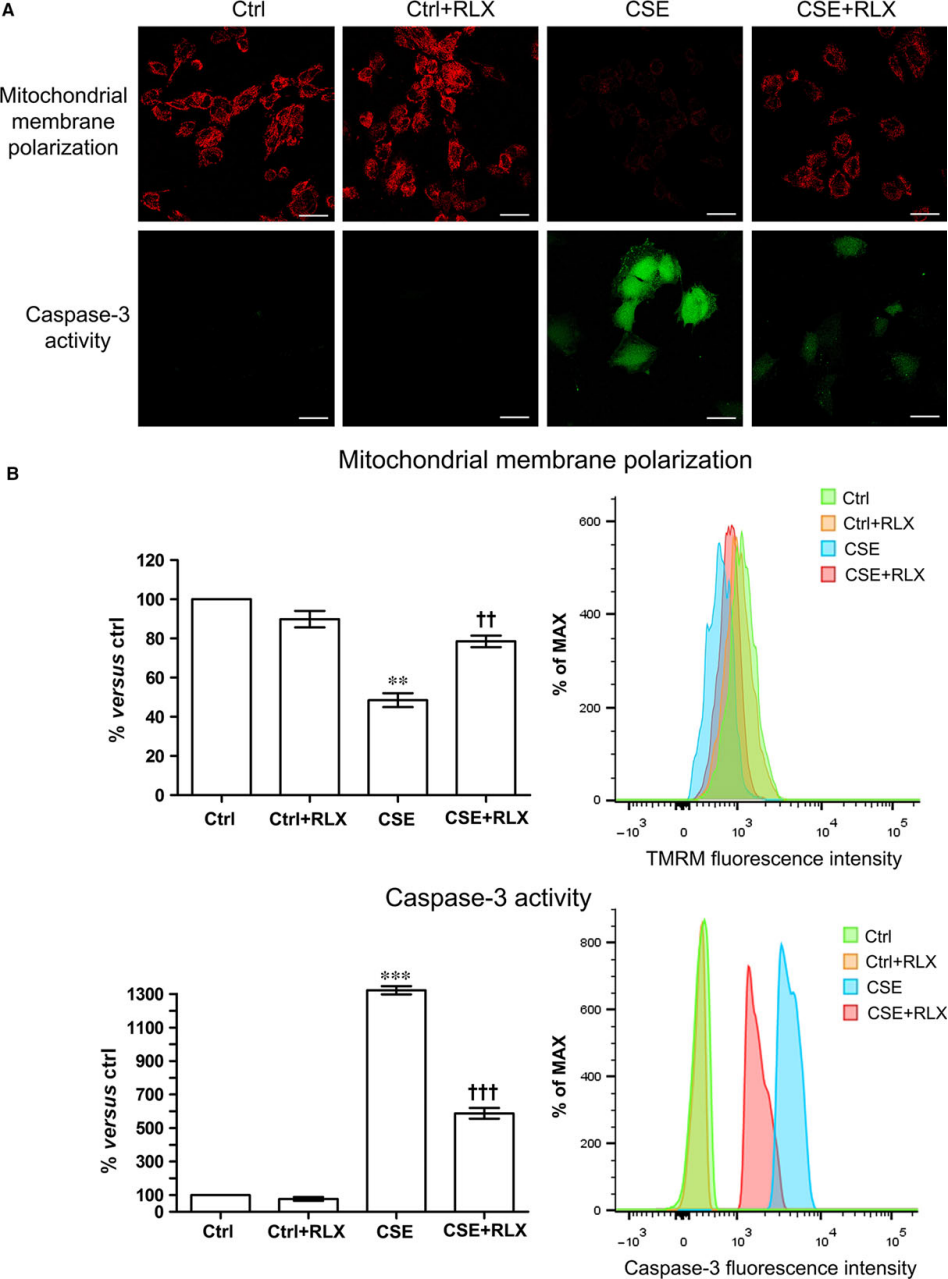
Evaluation of apoptosis in GPAE cells. (**A**) Confocal microscopy of GPAE cells subjected to TMRM and caspase‐3 activity assays. Exposure to CSE causes a marked reduction of mitochondrial membrane polarization and increase in caspase‐3 activity, which are counteracted by RLX. **(B)**. FACS analysis confirms the above findings. Values are means ± S.E.M. (one‐way anova test), *n* = 3. **P < 0.01, ***P < 0.001 *versus* control. ^††^ *P* < 0.01, ^†††^ *P* < 0.001 *versus* CSE. RLX *per se* has no pro‐apoptotic effect, bar: 20 μm.

To investigate the mechanisms of the protection afforded by RLX on the aortic endothelium upon exposure to CS, we performed a series of *in vitro* experiments on primary GPAE cells. These cells were first shown to express the RLX receptor RXFP1 (Fig. 4A). When exposed to CSE for 4 hrs, GPAE cells showed a marked viability decrease, as assayed with the trypan blue exclusion test. Pre‐treatment for 24 hrs with 100 ng/ml RLX significant reduced this adverse effect of CSE (Fig. 4B). Reduced cell viability by CSE was correlated with the induction of apoptosis. Exposure to CSE induced a prominent decrease in mitochondrial membrane polarization, an early apoptotic event, and increase in caspase‐3 activity, a marker of ongoing apoptosis, in comparison with the untreated controls (Fig. 5A and B). Incubation with RLX significantly reduced CSE‐induced apoptosis (Fig. 5A and B). Addition of RLX to untreated control cultures had no effect on cell viability and apoptosis.

### Endothelial oxidative injury

**Figure 6:**
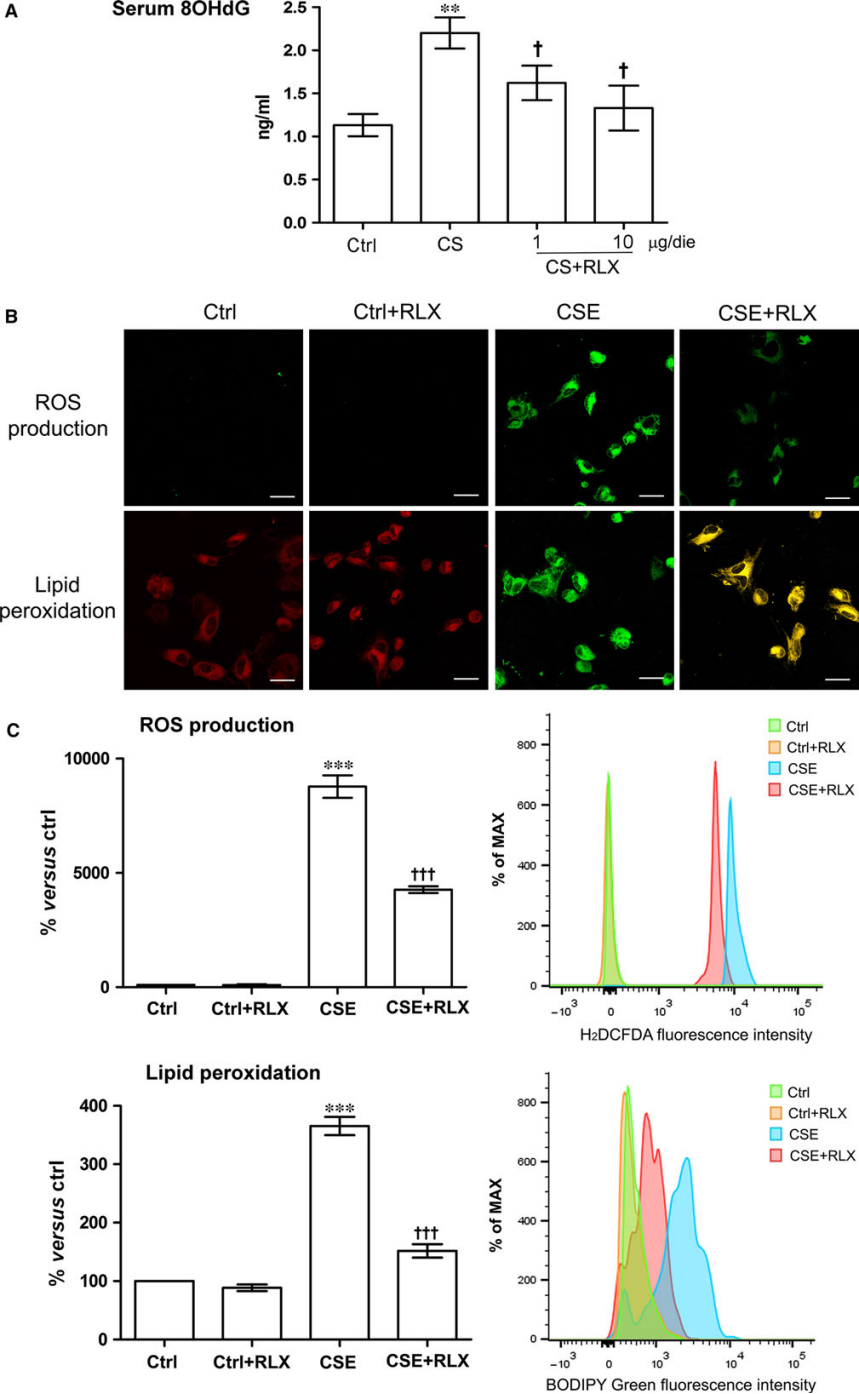
Evaluation of oxidative stress. (**A**) Levels of 8‐OHdG, a DNA oxidation marker, in the different groups of animals: compared with the controls, CS causes a significant increase in 8‐OHdG, which was reduced by RLX at both doses. Values are means ± S.E.M. (one‐way anova test), *n* = 6. **P < 0.01, *versus* control. ^†^ *P* < 0.05 *versus* CS. (**B**) Confocal microscopic evaluation of intracellular ROS generation and lipoperoxidation by H2DCFDA and BODIPY on GPAE cells: both these parameters are enhanced upon CSE treatment and significantly reduced by RLX. (**C**) FACS analysis confirms the confocal microscopic findings. Values are means ± S.E.M. (one‐way anova test), *n* = 3. ***P < 0.001 *versus* control. ^†††^ *P* < 0.001 *versus* CSE. RLX *per se* has no pro‐apoptotic effect, bar: 20 μm.

Because oxidative injury is a typical detrimental effect of CS (5‐10), we then investigated whether the observed endothelial damage was related to oxidative stress. In fact, the serum levels of 8‐OHdG, a marker of DNA oxidation, were significantly increased in the guinea pigs exposed to CS in comparison with the untreated controls, while administration of RLX at both doses significantly reduced serum 8‐OHdG (Fig. 6A). In keeping with these results, 4‐hr exposure of GPAE cells to CSE caused a robust increase in intracellular ROS (Fig. 6B), resulting in significantly higher membrane lipid peroxidation (Fig. 6C), as compared with the untreated controls. The treatment with RLX significantly reduced both these CSE‐induced oxidative stress markers (Fig. 6B and C). Addition of RLX to untreated control cultures had no effect.

### Endothelial NOS expression

**Figure 7:**
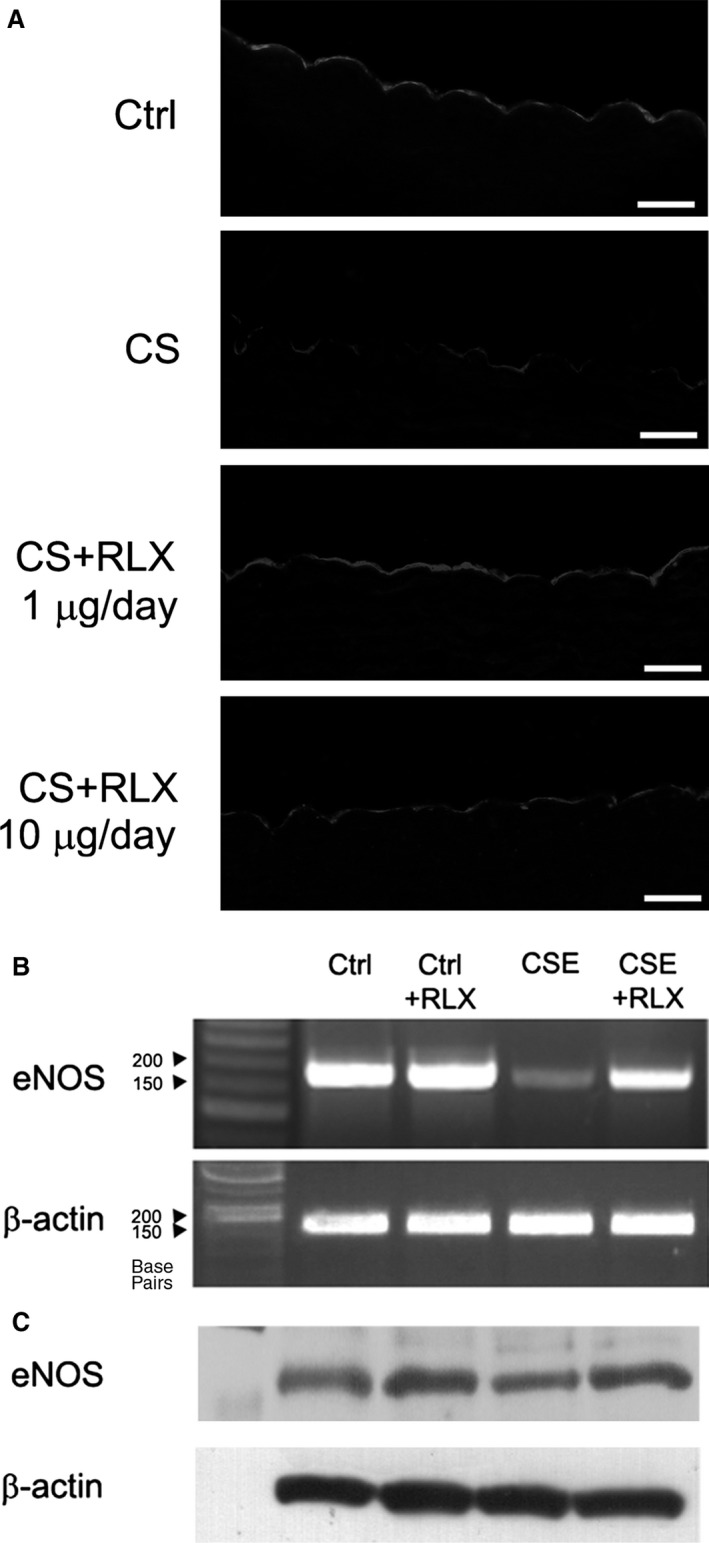
Expression of constitutive endothelial NOS (eNOS). (**A**) eNOS immunoreactivity in aortic sections from guinea pigs of the different experimental groups: compared with the control, CS causes a marked reduction of eNOS immunoreactivity in the endothelial lining, while this adverse effect is counteracted by RLX at both doses, magnification ×400, bars = 20 μm. Expression of eNOS mRNA (**B**) and protein (**C**) in GPAE cells evaluated by RT‐PCR and Western blotting, respectively. Compared with the controls, eNOS is reduced by CSE and maintained by RLX. RLX *per se* has no appreciable effects.

Cigarette smoke‐induced endothelial injury and dysfunction has been associated with impaired eNOS expression 14, 15, 16. Accordingly, the current *in vivo* experiment has shown that CS causes a well‐appreciable decrease in eNOS immunoreactivity in the residual endothelial cells of the aortic wall as compared with the untreated controls. This eNOS impairment was reduced in the animals treated with RLX at both doses (Fig. 7A). The supportive effect of RLX on eNOS expression was confirmed *in vitro*: in fact, eNOS mRNA (Fig. 7B) and protein expression (Fig. 7C) in GPAE cells were markedly reduced in the CSE‐treated cultures, while incubation with RLX significantly reduced the CSE‐induced eNOS reduction. Addition of RLX to untreated control cultures had no appreciable effect.

## Discussion

The results of the present study show that RLX protects against aortic dysfunction and endothelial impairment induced by CS. In fact, long‐term administration of RLX to guinea pigs exposed chronically to CS preserved the responsiveness of isolated aortic strips to phenylephrine and Ach, which was compromised by CS. Namely, the decreased amplitude of the vasodilatory responses to Ach accounts for impaired endothelial function, as also reported in previous experimental studies on animals exposed to chronic smoking 12. On the other hand, the impairment in contractile response to phenylephrine observed in the preparations from CS‐exposed animals is likely related to adverse arterial wall remodelling, a typical effect of CS‐induced vascular injury 4, 20. The improvement of the contractile response to phenylephrine induced by RLX treatment fits well with the present observation of preserved histology of the aortic wall and is consistent with the report that RLX therapy prevents and even reverses large artery remodelling and fibrosis in a rat model of chronic hypertension 50. In our experimental model, CS exposure and RLX treatment were concurrent, thus the reported findings support the notion that RLX prevents CS‐induced arterial injury and dysfunction but do not provide information to understand whether RLX treatment could revert established vascular damage because of CS.

The observed preservation of the vasodilatory property of aortas in the CS‐exposed guinea pigs which received RLX can be chiefly related to the maintenance of the endothelial lining and of adequate levels of eNOS in endothelial cells, as can be argued from the present *in vivo* and *in vitro* findings. This property of RLX fits well with previous reports that this hormone can up‐regulate NOS expression in vascular endothelial cells 26, 27, 28, 29, 30. However, RLX may also promote arterial dilatation by a direct effect on vascular smooth muscle cells, as reported 29.

Our study indicates that the dysfunctional vasodilatory response of the aortas in CS‐exposed guinea pigs is related to endothelial impairment because of oxidative stress, as suggested by the rise in serum 8‐OHdG. Indeed, in these animals, the aortic endothelium was lacking in large tracts of the intima and, when present, showed ultrastructural signs of cell injury and a remarkable reduction of eNOS expression. Consistently, exposure of GPAE cells to CSE caused a significant reduction of cell viability and induction of apoptosis, which were closely related to increase in intracellular ROS and lipid peroxidation. The present findings first show that RLX, at physiological circulating levels or nanomolar concentrations in the culture medium, protects endothelial integrity and function by reducing oxidative stress both *in vivo* and *in vitro*.

The cellular mechanisms accounting for this antioxidant effect remain to be elucidated. Preliminary findings from our laboratory obtained with the oxygen radical absorbance capacity assay indicate that RLX has no intrinsic antioxidant properties. On the other hand, RLX administration to GPAE cells markedly increased their endogenous antioxidant capacity: in the presence of CSE, RLX restores it to levels near to the untreated controls (Supporting Information). It can be speculated that RLX may up‐regulate one or more cellular defence mechanisms against oxidative stress through activation of PI3 and MAP kinases, typical downstream pathways of RXFP1 activation in blood vessels 30, 51. These signalling pathways are in fact involved in the cell response to ROS, being able to induce the phosphorylation of the transcription factor nuclear factor‐E2‐related factor 2 (Nrf2), which binds the antioxidant response element within the promoters of genes encoding antioxidant and detoxifying enzymes 52. This hypothesis for the mechanism of action of RLX on cell resistance to oxidative stress deserves further investigation.

A limitation of this study is that we did not explore *in vivo* physiological parameters, such as blood pressure or pulse pressure, useful to further assess RLX's therapeutic effects. However, the current observation that RLX is capable of reducing CS‐mediated vascular damage and dysfunction may be relevant to human health. In fact, it is known that post‐menopausal women who are smokers have double the risk of CVD, a discrepancy which has been related to defective ovarian hormones 53. However, the protection afforded by hormone replacement therapy (HRT) with oestrogen/progestin is controversial, as some clinical trials failed to confirm that HRT can be beneficial. Two randomized prospective primary or secondary prevention trials showed that HRT may actually increase the risk and events of CVD in post‐menopausal women 54. The failure of HRT in lowering the cardiovascular risk of smoking in post‐menopausal women can be explained considering that cessation of ovarian function at menopause implies not only a drop in the secretion of oestrogen and progesterone but also of H2 relaxin, which is mainly secreted by the corpus luteum 55 and has been credited of major protective activities on the cardiovascular system 23, 29, 56. On this ground, RLX could be viewed as a new therapeutic tool to prevent or reduce the cardiovascular risk in smokers: this hypothesis is strengthened by the successful use of RLX in the treatment of patients with heart failure demonstrated in a recent clinical trial, which also revealed that the use of RLX in humans is safe and free of adverse side effects 24, 57.

## Conflict of interest

The authors declare that they have no conflict of interest.

## Supporting information


**Data S1** Oxygen radical absorbance capacity (ORAC) assay.
