# Supplementary material for: Protection from cigarette smoke‐induced vascular injury by recombinant human relaxin‐2 (serelaxin)
Source: J Cell Mol Med. 2016 Feb 24;20(5):891–902. doi: 10.1111/jcmm.12802 (PMC4831370; doi:10.1111/jcmm.12802)
Supplement: Supplementary file 1 — Data S1 Oxygen radical absorbance capacity (ORAC) assay. [file JCMM-20-891-s001.doc]

**Oxygen Radical Absorbance Capacity (ORAC) Assay**

The antioxidant capacity was evaluated by the ORAC assay, following the protocol described previously (1). Briefly, the reaction was carried out in 96-well black microplates (Nunc, Roskilde, Denmark), with Trolox (0–200 μM) used as a standard. The working solution was obtained by combining 100 μl fluorescein solution (10 nM) in phosphate buffer (75 μM, pH 7.4). Then, 70 μL of each analyzed sample in PBS was added (10 μg of protein) and incubated for 30 min at 37 °C. The reaction started with 50 μl of pre-heated (at 37°C) free radical generator AAPH (Sigma-Aldrich Italy S.r.l.) solution (final concentration 200 mM). Fluorescence was recorded kinetically for 3 hours at 37 °C using a fluorometric microplate reader (Fluoroskan Ascent; Thermo Electron Corp., Vantaa, Finland) at excitation and emission wavelengths of 485 and 537 nm, respectively. All assays were conducted in triplicate and at least three independent tests were carried out for each sample. The area under curve was calculated for each sample by integrating the relative fluorescence curve. Regression equations obtained from net value of Trolox were used to calculate the ORAC value for each assay. Final ORAC values were expressed as nmol Trolox equivalent (TE) per mg of protein (nmol TE/mg).

Control: 617.9 nmol TE/mg

Control+RLX: 1156.4 nmol TE/mg

CSE: 538.3 nmol TE/mg

CSE+RLX: 597.8 nmol TE/mg

RLX molecule has no intrinsic antioxidant capacity (0.002 nmol TE/mg protein)

**1. Barygina VV, Becatti M, Soldi G, Prignano F, *et al*.** Altered redox status in the blood of psoriatic patients: involvement of NADPH oxidase and role of anti-TNF-α therapy. *Redox Rep*. 2013; 18:100-6.
